# Dynamics of dental evolution in ornithopod dinosaurs

**DOI:** 10.1038/srep28904

**Published:** 2016-07-14

**Authors:** Edward Strickson, Albert Prieto-Márquez, Michael J. Benton, Thomas L. Stubbs

**Affiliations:** 1 School of Earth Sciences, University of Bristol, 24 Tyndall Avenue, Bristol BS8 1TQ, UK

## Abstract

Ornithopods were key herbivorous dinosaurs in Mesozoic terrestrial ecosystems, with a variety of tooth morphologies. Several clades, especially the ‘duck-billed’ hadrosaurids, became hugely diverse and abundant almost worldwide. Yet their evolutionary dynamics have been disputed, particularly whether they diversified in response to events in plant evolution. Here we focus on their remarkable dietary adaptations, using tooth and jaw characters to examine changes in dental disparity and evolutionary rate. Ornithopods explored different areas of dental morphospace throughout their evolution, showing a long-term expansion. There were four major evolutionary rate increases, the first among basal iguanodontians in the Middle-Late Jurassic, and the three others among the Hadrosauridae, above and below the split of their two major clades, in the middle of the Late Cretaceous. These evolutionary bursts do not correspond to times of plant diversification, including the radiation of the flowering plants, and suggest that dental innovation rather than coevolution with major plant clades was a major driver in ornithopod evolution.

Ornithopod dinosaurs represent one of the most successful radiations of Mesozoic vertebrates^1^, showing several steps in diversification through the Jurassic and Cretaceous (Fig. 1). The clade consisted of obligatory to facultative bipedal herbivores ranging from small (1–2 m in length) cursorial to larger (over 12 m in length) animals^1^,^2^,^3^. Ornithopods colonised every continent and left a rich fossil record spanning from the Middle Jurassic^4^ to the latest Cretaceous^5^. Most diverse and abundant were the specialized ‘duck-billed’, and often crested, hadrosaurids. These ornithopods constitute one of the most common and diverse megaherbivore clades of the Campanian–Maastrichtian (latest Cretaceous) of Eurasia, the Americas and Antarctica^6^.

The feeding apparatus has been identified as key in the evolution of ornithopod dinosaurs, allowing them to master a broad range of herbivorous diets^7^. Ornithopod dentitions showed dramatic changes throughout their evolution. The most basal ornithopods, exemplified by *Orodromeus mackelai*^8^ and jeholosaurids like *Haya griva*^9^ and *Changchunsaurus parvus*^10^ have relatively low tooth counts, ranging from 14–17 alveolar positions. These early ornithopods have low tooth crowns that are labiolingually compressed, basally constricted, bulbous to subtriangular in shape, and ornamented with numerous apicobasal ridges. Marginal denticles in these forms are typically large and triangular.

The most basal iguanodontians, such as rhabdodontids^5^,^11^, also have low tooth crowns, but these become larger and more elongate, acquiring a more lanceolate profile while preserving numerous ridges and showing a prominent median carina. More derived iguanodontians such as *Camptosaurus dispar*^12^, *Kukufeldia tilgatensis*^13^, *Lanzhousaurus magnidens*^14^ and *Tenontosaurus tiletti*^15^ have a slightly higher tooth count, approaching 20 alvelolar positions, and shield-like tooth crowns bearing ridges that tend to be shorter and restricted to the apex and margins of teeth. A few of these ridges are more prominent and a larger median carina is still present^1^,^16^.

The key explosion in ornithopod diversity was the expansion of hadrosaurids in the Late Cretaceous (Fig. 1). These account for about 40% of known ornithopod species^6^ and 30–80% of individual specimens in many latest Cretaceous dinosaur communities of North America and Europe^17^,^18^. Hadrosaurid teeth are characterised by the reduction of ridges to a single median carina, accompanied in the hollow-crested lambeosaurines by one or two subsidiary ridges, further elongation of lanceolate tooth crowns that were at least twice as tall as wide, absence or decrease in the size of marginal denticles, tooth miniaturisation, and a substantial increase in the number of alveolar positions, reaching the extreme of nearly 60 positions in Edmontosaurini^19^, and increase in the width of the occlusal surface to a maximum of three teeth arranged labiolingually^6^,^20^,^21^,^22^,^23^.

Key to understanding evolutionary dynamics is the balance of biotic and abiotic factors, whether these herbivores owed their success primarily to evolutionary innovation or to external drivers such as the evolution of new plant types^24^. In particular, was the radiation of hadrosauroids in the latest Cretaceous triggered by the Cretaceous Terrestrial Revolution (KTR)^25^. Between 125 and 80 Ma, the diversity of terrestrial animals expanded substantially alongside the evolution and success of angiosperms^26^. This event is associated with the evolution of eusocial insects and grasses, and coincides broadly with the diversification of various dinosaurian herbivores, including neoceratopsians and hadrosauroids^25^. The impact of angiosperm evolution on herbivorous dinosaurs has been queried^25^,^27^. Since the KTR changed the available vegetation in the Late Cretaceous, and therefore the available ecological niches that hadrosaurids could have exploited, its influence on ornithopod evolution should be considered.

Here, we provide a quantitative macroevolutionary perspective on the evolutionary dynamics of ornithopod dentition. In particular, how influential were those character transformations during ornithopod evolution? Is there a trend towards increasing disparity throughout ornithopod dental evolution? Are the distinct tooth morphologies seen throughout ornithopod evolution supported by significant changes in disparity? Do shifts in evolutionary rate precede or coincide with the evolution of these morphologies? Is there a correlation between evolutionary rate and disparity in ornithopod tooth evolution? Were ornithopod dentitions influenced by external drivers such as the evolution of new plant groups, and most notably the KTR?

## Results

### Morphospace occupation

There is clear evidence of an evolutionary gradient in ornithopod dental morphospace (Fig. 2a,b). Basal taxa cluster in the lower left quadrant, entirely separate from hadrosaurids and their more closely related hadrosauroid outgroups. An intermediate grade, incorporating basal ankylopollexians, dryosaurids and basal styracosterns, bridges the gap between basal ornithopods and hadrosauroids. Overall, styracosternan taxa occupy the majority of the morphospace, and they retain the same position through the Jurassic and Cretaceous. Hadrosaurids cluster in a densely packed region of morphospace (Fig. 2a,b), implying similar dental morphologies. Their predecessors among hadrosauroids and Iguanodontia *sensu lato* occupy wider morphospaces. This suggests that early hadrosauroids explored an array of morphologies before focusing on the reduced hadrosaurid morphospace seen in lambeosaurines and saurolophines. The distinction of the morphospaces occupied by the four morphogroups (Fig. 2b) is confirmed by PERMANOVA tests (Supplementary Table 1).

Temporal morphospace plots illustrate the diversification of ornithopod dentitions from the Late Jurassic to the end-Cretaceous (Fig. 2c). The overall area of morphospace occupied shows both expansions and contractions between successive intervals. Dental morphospace occupation is consistent from the Hauterivian to the Turonian. The last three Cretaceous bins show the large diversfication of the hadrosaurids. Interestingly, this new hadrosaurid morphotype is concentrated in the right-hand quadrants, but collectively it does not massively expand the overall ornithopod morphospace, despite the great numerical dominance of species. After their origin and expansion, the hadrosaurid morphospace remains static and consistent in the Campanian and Maastrichtian. The largest morphospace occupation occurs in the Maastrichtian, with hadrosaurids and basal ornithopods at opposite ends. PERMANOVA tests confirm that morphospace occupation in the Campanian and Maastrichtian was largely distinct from that in the Kimmeridgian to Turonian interval, while the Coniacian–Santonian was a transitional bin (Fig. 2c; Supplementary Table 2).

### Disparity and diversity trends

Taxon-binned disparity metrics (Fig. 3) show similar results for generalised Euclidean distances (Fig. 3a) and maximum observable distances (Fig. 3b). Despite their great diversity surge in the Campanian–Maastrichtian, hadrosaurids do not show a significantly greater disparity than other groupings. Instead, non-hadrosaurid hadrosauroids display the highest disparity in each case, followed by the rhabdodontids (although these have the largest error bars). Next in disparity are basal iguanodontians and hadrosaurids as a whole; among the latter, the hollow-crested lambeosaurines show a relatively high disparity. The sum of variance metrics (Fig. 3c) place basal iguanodontians generally low compared to hadrosaurids. The solid-crested/unadorned saurolophine hadrosaurids consistently show lower disparity than lambeosaurines, and generally lower disparity than all but the most basal ornithopods.

Two diversity metrics indicate that ornithopod diversity (Fig. 4a,b) rises gently during the Aptian and Albian, then dips, and subsequently peaks in the Campanian. Disparity (Fig. 4c–e) shows a rather different pattern, with a dip in the Berriasian–Valanginian, followed by a rise to a peak in the Coniacian–Santonian. Disparity then hits a trough in the Campanian before rising in the Maastrichtian. This Campanian trough coincides with the hadrosaurid diversity surge (Fig. 4a,b), showing the Campanian as the point of highest taxic and lineage diversity, but with surprisingly low disparity. This trend is consistent when the sum of variances is calculated with different numbers of PC axes (Supplementary Fig. 2). In addition, the Campanian also matches the peak of evolutionary rates (Fig. 4f), with the highest per-lineage-million-years rates of character change.

### Evolutionary rates

Significantly elevated evolutionary rates are consistently identified on four internal branches of the phylogeny (Fig. 5). These instances of fast evolution correspond to the emergence of iguanodontians more closely related to rhabdodontids than to *Anabisetia*, the split of lambeosaurines and saurolophines within hadrosaurids, the origin of saurolophines, and the origin of a more derived lambeosaurine clade, including those taxa more closely related to *Charonosaurus* than to *Tsintaosaurus* and *Pararhabdodon*. Many terminal branches show accelerated rates, primarily within hadrosaurids and basal iguanodontians. At the base of the tree, *Orodromeus,* the most basal ornithopod, shows decreased rates, along with other basal ornithopods like *Thescelosaurus* and *Haya*, and the rhabdodontid *Rhabdodon*. Rates results are generally consistent across all most parsimonious trees (MPTs), although minor differences are seen in the percentage of iterations that are significant/insignificant (Supplementary Figs 3–6).

**Figure 1:**
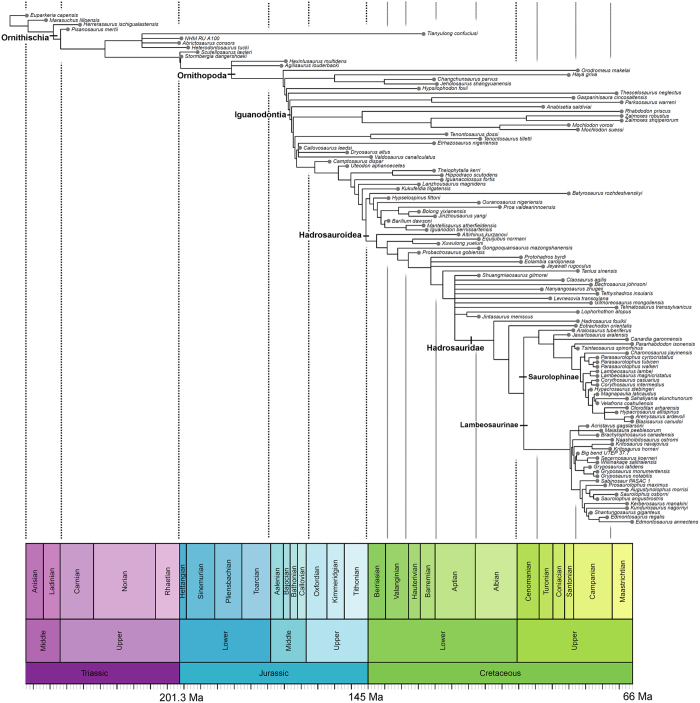
Supertree of Ornithopoda. This species-level phylogeny is based on maximum parsimony analysis of a matrix representation with parsimony analysis, integrating several recent phylogenetic hypotheses^9^,^38^,^39^,^40^,^41^ resolving the relationships of major ornithopod clades. Each taxon is plotted stratigraphically based on its first appearance date.

The timings of these diversification shifts are telling. The first happened minimally in the Middle–Late Jurassic, and the other three, possibly close together, in the Turonian and Coniacian, about 90 Ma (cf. Figs 1 and 5). Time series plots of evolutionary rates (character changes per lineage; Fig. 4f) also illustrate marginally higher rates in the earliest sampled bin (Kimmeridgian–Tithonian), followed by a long interval of consistently slow rates, before a large increase in evolutionary rate from the Coniacian to Maastrichtian. Therefore, the first evolutionary acceleration long predated the KTR, and the final three substantially post-dated the initiation of the radiation of angiosperms, in the Early Cretaceous, around 125–120 Ma. Admittedly, angiosperms continued diversifying and expanding in abundance within ecosystems throughout the Cretaceous, and they showed several dramatic diversification shifts from 90–40 Ma^28^.

## Discussion

**Figure 2:**
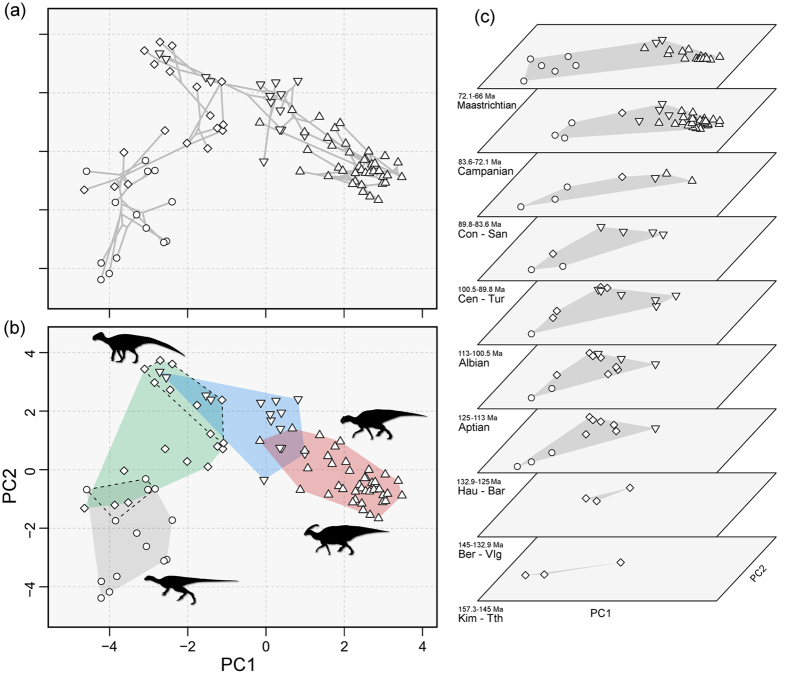
Ornithopod dental morphospaces. All plots are dental morphospaces defined by PC1 (8.5%) and PC2 (4.2%). (**a**) Phylomorphospace showing the distribution of 89 ornithopod species in dental morphospace and the branching pattern from the supertree. (**b**) Morphospace plot representing the same data, but with four evolutionary grades highlighted: basal Ornithopoda (grey), an intermediate grade of ankylopollexian, dyrosaurid and basal styracostern species (green), non-hadrosaurid hadrosauroids (blue), and hadrosaurids (red). Basal rhabdodontids (lower) and intermediate basal styracosterns (higher) are highlighted using discontinuous lines. (**c**) Distribution of ornithopods in dental morphospace in nine time intervals, ranging from the Kimmeridgian to the Maastrichtian. The overall area of morphospace occupied by all taxa is highlighted with a shaded convex hull. A plot with each taxon individually labelled is presented in Supplementary Figure 1. Stratigraphic abbreviations: Bar, Barremian; Ber, Berriasian; Cen, Cenomanian; Con, Coniacian; Hau, Hauterivian; Kim, Kimmeridgian; San, Santonian; Tth, Tithonian; Tur, Turonian; Vlg, Valanginian. Silhouettes were drawn by Pete Buchholz, Scott Hartman and Jaime Headden, and were downloaded from http://phylopic.org.

Our study confirms some long-held understandings of ornithopod evolution, and rejects others. The massive ecological impact of hadrosaurids on Late Cretaceous ecosystems is confirmed by their explosive diversification (Figs 1 and 4a,b), and there is a definite substantial shift in morphospace occupation, confirming different feeding adaptations than their precursors (Fig. 2). However, the remarkably tight clumping of all hadrosaurids in morphospace (Fig. 2c) suggests that, once their extreme dental adaptations had emerged, little changed, and that their dietary specialisation was successful over wide geographic areas and through time.

This is reflected also in the statistically significant dip in disparity in the Campanian (Fig. 4c–e), accompanied by a peak in ornithopod diversity and evolutionary rate, indicating substantial numbers of species that all adopted a limited array of morphologies of jaws and teeth. Not only were these Campanian hadrosaurids diverse, they were also abundant, within ecosystems in North America and Mongolia, attesting to their mastery of a successful herbivorous dietary adaptation.

**Figure 3:**
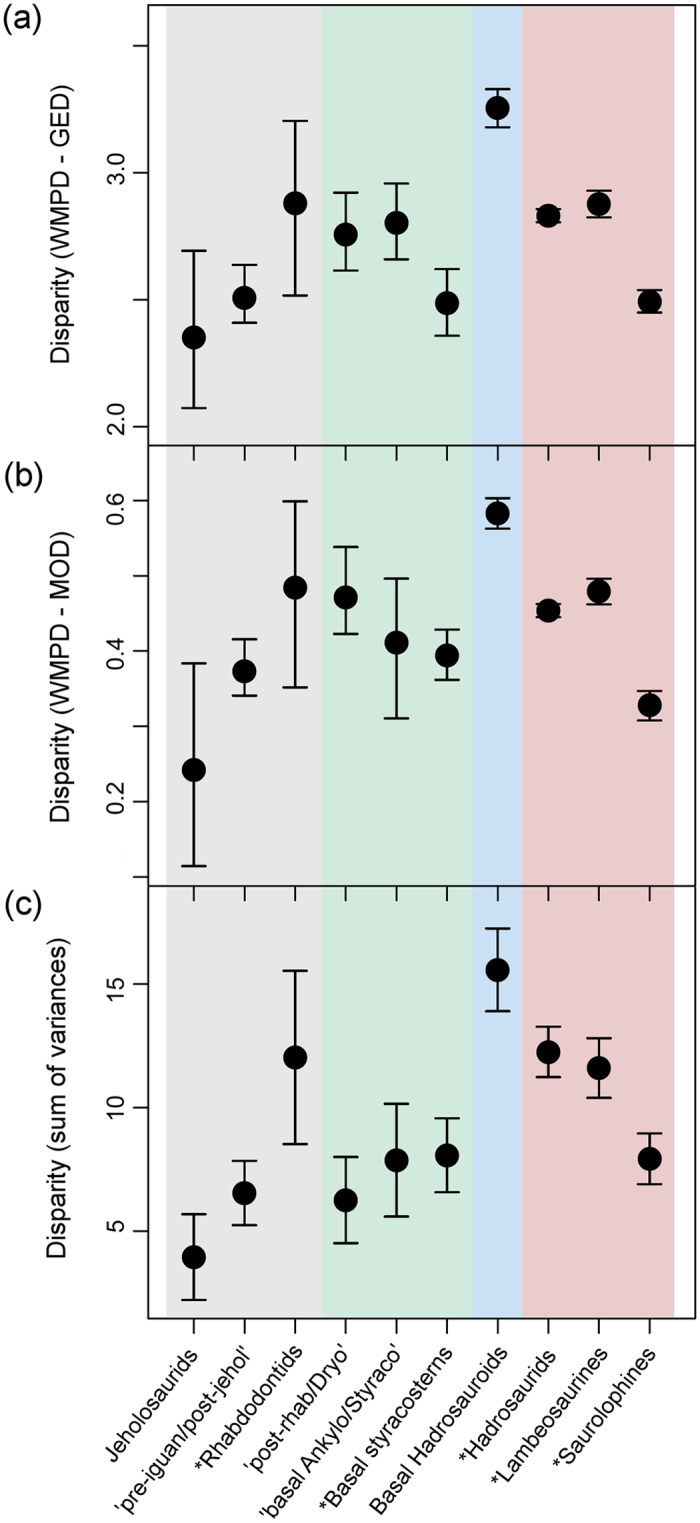
Ornithopod dental disparity. Disparity results based on taxa binned in ten taxonomic groups. Disparity values based on the weighted mean pairwise disparity (WMPD) from the generalised Euclidean distance matrix (GED) (**a**), the maximum observable distance matrix (MOD) (**b**), and the sum of variances from PCO scores (**c**). 95% confidence intervals based on 10,000 bootstrap replicates are also plotted for each metric. Monophyletyic groups are denoted with asterisks. The abbreviated taxonomic assemblages are: ‘pre-iguanodontids and post‐jeholosaurids’, ‘basal ankylopollexians and basal styracosterns’, and ‘post-rhabdodontids and dryosaurids’. Background colour shading corresponds to the evolutionary grades in Fig. 2.

Despite the statistical significance of the differences between hadrosaurid morphospaces and those of other ornithopods, it should be noted that lambeosaurine disparity was much higher than saurolophine disparity (Fig. 3). In addition, both clades experienced significant rate increases after the initial significant rate increase that preceded their divergence (Fig. 5).

Significantly slow rates of evolution were found in basal ornithopods from the Late Cretaceous, such as *Orodromeus* (Fig. 5). Since these organisms retained so many basal characteristics so long after they evolved, it is no surprise that they are associated with slower evolutionary rates. Comparative study of overall dinosaurian evolution^25^,^29^ confirms that the bulk of diversification happened in the Late Triassic and Early Jurassic. Only two clades showed significantly rapid diversification in the Cretaceous, the hadrosaurids and neoceratopsians^25^. Our finding that hadrosaurids and hadrosauroids show several accelerations in evolutionary rate, confirms these earlier conclusions.

It has been proposed that the origin of hadrosaurids, along with the origin of ceratopsids, both of which possessed dental batteries^30^, was influenced by the KTR^31^. These suggestions have been discounted^24^,^25^, and our results confirm this, in that the timings do not match. Perhaps hadrosaurids did consume some angiosperms^32^, and the uniquely high number of dental tissues^22^ in hadrosaurids could have permitted them to process several vegetation types. However, there is little evidence that hadrosaurids became angiosperm specialists – in fact, their grinding dental batteries, tooth microwear, stomach contents and coprolites indicate that they were conifer specialists^33^. In addition, the nearly cosmopolitan geographic range of hadrosaurids includes occupation of areas with few angiosperms, such as high latitude zones^34^. Therefore, in line with previous studies^24^,^25^,^27^,^35^, we find little evidence that dinosaurian herbivore evolution was primarily controlled by the varying fates of major plant groups through the Mesozoic.

**Figure 4:**
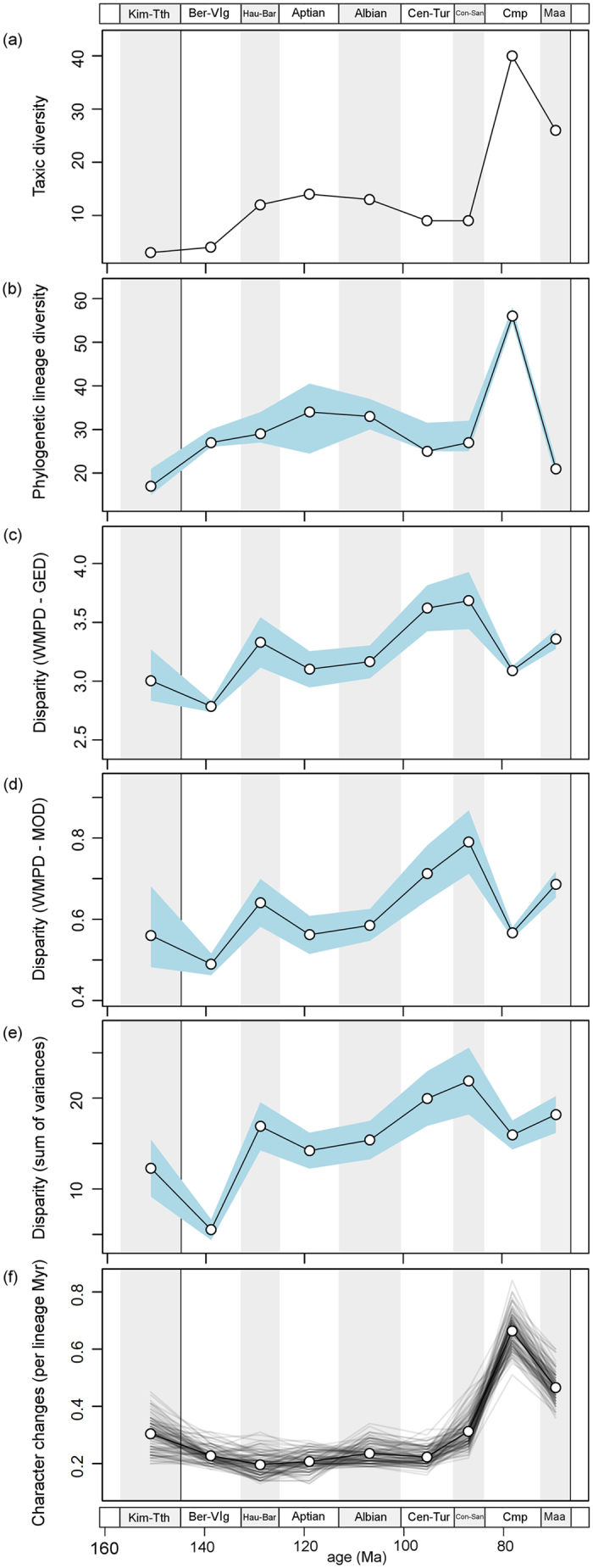
Temporal trends of ornithopod diversity, disparity and evolutionary rates. Taxic diversity (**a**) and phylogenetic lineage diversity (**b**) are plotted in nine time intervals ranging from the Kimmeridgian to the Maastrichtian. In (**b**), the median diversity is plotted from 100 topologies along with confidence envelopes representing two-tailed 95% lower and upper quantiles. Disparity is plotted (white circles) in the same nine time intervals, based on weighted mean pairwise disparity (WMPD) from the generalised Euclidean distance matrix (**c**), the maximum observable distance matrix (**d**), and the sum of variances from PCO scores (**e**). The blue envelopes in (**c–e**) represent 95% confidence intervals based on 10,000 bootstrap replicates. Plot (**f**) illustrates the temporal variation in evolutionary rates based on the mean number of character changes per lineage million years within the nine bins. Each line represents rates results from the 100 analysed topologies and the mean rate per bin is plotted (white circles). Stratigraphic abbreviations as for Fig. 2, plus Cmp, Campanian; Maa, Maastrichtian.

**Figure 5:**
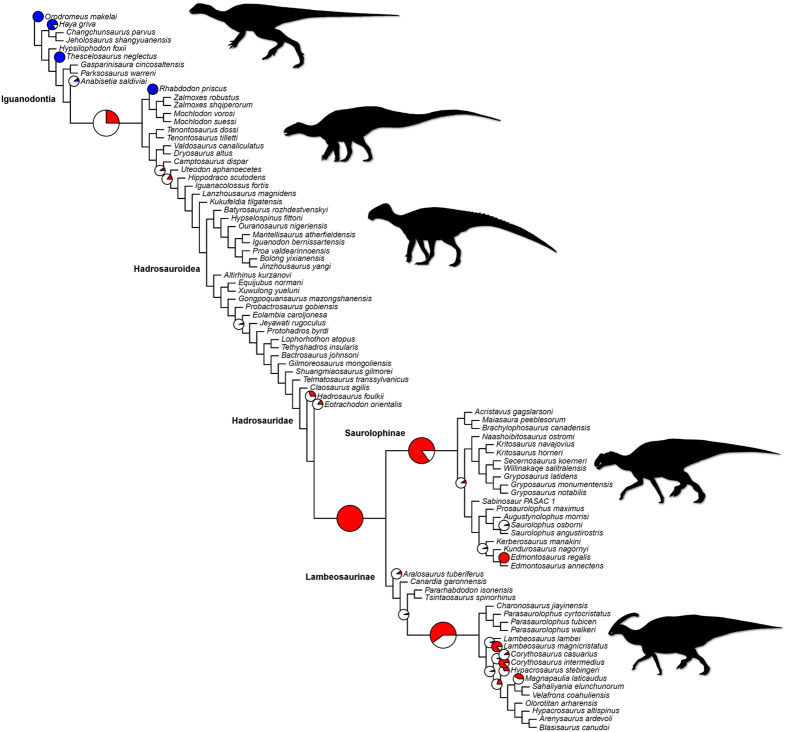
Rates of morphological evolution based on dental characters in Ornithopoda. The proportion of significantly high (red) and significantly low (blue) per-branch rates based on 20 dating replications are illustrated with pie charts. The topology figured is one randomly selected MPT (MPT 163) from the 190 MPTs recovered in the supertree analyses. Four additional topologies are figured in the supplement. There are no pie charts positioned on branches that showed nonsignificant rates in all dating replicates. Four internal branches that showed significantly high/low rates in more than 25% of trees are lengthened and have enlarged pie charts. Silhouettes were drawn by Pete Buchholz, Scott Hartman and Jaime Headden, and were downloaded from http://phylopic.org.

In our study, we find that diversity and disparity are decoupled – compare the offset peaks in both metrics (Fig. 4), in which ornithopod dental disparity peaked in the Coniacian-Santonian, but diversity peaked in the Campanian. Whether this can be said to represent an example of a ‘disparity-first’ model, the commonest finding in comparative macroevolutionary studies^36^, is unclear. Nevertheless, our study confirms the common observation that diversity and disparity are decoupled, each varying according to its own drivers, an important general observation in macroevolution^29^,^36^.

Our work confirms the significance of tooth morphology and the emergence of the hadrosaurid dental battery during the evolution of iguanodontian ornithopods through distinctive shifts in morphospace occupation and evolutionary rates. These findings would benefit from additional data on the non-dental anatomy of the organisms studied, to add context and compare the significance of dental evolution to other important adaptations of hadrosaurids, such as the evolution of hypertrophied nasal passages and cranial ornamentation. Even so, the explosion of hadrosaurid diversity in the Campanian and Maastrichtian, at a time when overall ornithopod disparity was low, suggests that the morphology of the hadrosaurid feeding apparatus changed little once established in both the lambeosaurine and saurolophine clades. Hadrosaurids were some of the most diverse, abundant, and geographically widespread terrestrial tetrapod herbivores ever. Their unique dental battery, adapted for efficient handling of tough vegetation, seems to have been the key to their diversity explosion rather than any particular vegetational changes.

## Materials and Methods

The importance of the hadrosaurid dentition and feeding mode on their high diversity in the Late Cretaceous is emphasized by the fact that this clade and neoceratopsians appear to buck the trend of overall dinosaur decline through the last 40 million years of the Cretaceous, recently discovered from Bayesian analysis of overall dinosaurian speciation dynamics^37^.

### Supertree

A species-level phylogenetic tree of Ornithopoda was constructed using source phylogenetic trees from the literature that resolve the relationships of major ornithopod subclades. Five recent trees were selected representing the scope of ornithopod diversity: Butler *et al.* (2011: Fig. 9)^38^, which resolves the position of basal ornithopods and provides a phylogenetic context for ornithopods within Ornithischia; Makovicky *et al.* (2011: Fig. 7B)^9^, which focuses on some of the more basal ornithopod subclades; Norman (2015: Fig. 51)^39^ and McDonald (2012: Fig. 1)^40^, which deal with the relationships of basal iguanodontians; and the recent hadrosaurid phylogeny by Prieto-Márquez (2016: Fig. 2)^41^. In addition, the rhabdodontid section of the phylogeny by Ősi *et al.*^11^ was added to the tree by Butler *et al.*^38^ to ensure full representation of this clade. Higher ornithischian outgroups to Ornithopoda taxa were replaced by one or two species, and ceratopsians were removed from the tree. *Euparkeria* was chosen as the most distant outgroup to Ornithopoda^42^.

Each source tree was manually drawn in Mesquite (version 3.02)^43^ and subsequently input into the Supertree Toolkit^44^ for calculation of a supertree using matrix representation with parsimony (MRP). The MRP was subjected to maximum parsimony analysis using TNT 1.1 (ref. 45). The tree was produced using 10,000 replicates with the tree bisection and reconnection algorithm, due to the size of the dataset and efficiency of the search method^46^. This analysis resulted in 190 MPTs of 177 steps each, an optimum score found 676 times out of the 10,000 replicates. A strict consensus tree was computed from these MPTs (Fig. 1).

### Dental characters

We scored 44 discrete dental morphological characters for 112 ornithopod and outgroup taxa (Supplementary Appendix 1). The traits represent dentary, maxillary, and, to a lesser extent, premaxillary teeth (absent in more derived ornithopods^47^). We chose these attributes as they document ornithopod feeding modes and the scope of morphological variation in these animals^25^. Specifically, 23 characters were culled from a character-taxon matrix that focused on hadrosaurids^6^, and eight and 13 were taken respectively from two studies^38^,^39^ that focused on non-hadrosaurid ornithopods. Taxon scoring was based on high-resolution photographs of fossil specimens, or using data from the scientific literature when photographs were not available, with a preference towards holotypes. The ontogeny of specimens was also recorded where available to ensure all specimens used were mature, and therefore comparable.

### Disparity analysis

To ensure that our data were compatible with principal coordinate analysis (PCO) and other disparity analyses, taxa with excessive missing data (e.g. solely known from postcranial remains or lacking teeth) were removed from consideration: *Callovosaurus, Elrhazosaurus, Barilium, Tanius, Levnesovia, Nanyangosaurus, Jintasaurus, Jaxartosaurus,* the Big Bend UTEP 37.7 hadrosaurid, *Shantungosaurus,* and *Theiophytailia*. Furthermore, all non-ornithopods were removed from the analysis.

The remaining taxa were placed in taxon-sorted and time-sorted bins. Taxon-sorted bins were based on the position of the taxa in the supertree, keeping monophyletic groups together where possible and avoiding bins with too few taxa to be representative. Five monophyletic clades were considered: jeholosaurids, rhabdodontids, hadrosaurids, saurolophine hadrosaurids and lambeosaurine hadrosaurids. However, keeping jeholosaurids as a taxon bin would leave the most basal ornithopod, *Orodromeus,* in a bin by itself. *Orodromeus* was therefore added to the ‘jeholosaurid’ bin. Taxon-sorted bins included: ‘jeholosaurids’, ‘pre-iguanodontids and post-jeholosaurids’, ‘rhabdodontids’, ‘post-rhabdodontids and dryosaurids’, ‘basal ankylopollexians and basal styracosterns’, ‘basal styracosterns’, ‘basal hadrosauroids’, ‘hadrosaurids’, ‘lambeosaurines’, and ‘saurolophines’. Some taxa that are traditionally placed within hadrosauroids are a part of ‘basal styracosterns’ rather than ‘basal hadrosauroids’, based on their position within the supertree.

The time-sorted bins represent ~10–15 Ma each and were based on first appearances of taxa according to the Paleobiology Database (https://paleobiodb.org). Five bins contain two stratigraphic stages, in order to equalise the numbers of taxa in each bin as much as possible and avoid unrepresentative bins with few taxa. The time bins are: Kimmeridgian-Tithonian (157.3–145 Ma), Berriasian–Valanginian (145–132.9 Ma), Hauterivian–Barremian (132.9–125 Ma), Aptian (125–113 Ma), Albian (113–100.5 Ma), Cenomanian–Turonian (100.5–89.8 Ma), Coniacian–Santonian (89.8–83.6 Ma), Campanian (83.6–72.1 Ma), and Maastrichtian (72.1–66 Ma).

Temporal and taxonomic disparity trends were assessed with two approaches. Firstly, the dental character-taxon matrix was used to calculate pairwise dissimilarity matrices based on generalised Euclidean distances (GED) and maximum observable distances (MOD), in the R package Claddis^48^. Morphological disparity in temporal and taxonomic bins was then calculated based on within-bin weighted mean pairwise disparity (WMPD), using both distance metrics. WMPD calculations place greater weighting on pairwise dissimilarities derived from more comparable characters, therefore preventing highly incomplete specimens from inflating or underestimating disparity^49^. In our second approach, we performed disparity calculations based on the positions of taxa in multivariate morphospace. The GED dissimilarity matrix was subjected to PCO to generate multivariate axes of variation, incorporating the Calliez negative eigenvalue correction. Disparity through time and within group bins was then quantified with binned PCO scores from the first 10 axes, using the sum of variances metric. Sensitivity tests were performed using different numbers of axes. In all disparity calculations, 95% confidence intervals were generated based on 10,000 bootstrap replicates.

To explore the morphological diversification of ornithopod dentitions, we examined taxon distribution in an empirical multivariate morphospace. The dental morphospace was based on a bivariate plot of PC axes 1 and 2, derived from the PCO described above. By plotting all taxa in a single pooled morphospace, we examined taxonomic trends and group separation. To explore the phylogenetic branching patterns within the dental morphospace, a pruned phylogeny with branch lengths (more below) was superimposed, producing a phylomorphospace. Temporal morphospaces were generated for the same bins as those used in the disparity analyses, and convex hulls denote the overall area of morphospace occupied through time.

Permutation tests were implemented to test for taxonomic and temporal separation in morphopace. One-way PERMANOVA multivariate statistical tests were performed on the PCO scores for both time and taxa-sorted bins, to test for statistical significance in multivariate group means^50^, with a chosen alpha level of 0.05. The results were adjusted using Benjamini-Hochberg corrections^51^ to reduce bias from multiple comparisons^52^.

### Evolutionary rates analysis

Rates of dental character evolution were examined with maximum-likelihood methods. All rate calculations were performed using the DiscreteCharacterRate function in the R package Claddis^48^. Our analyses aimed to determine if rates of dental evolution, based on the same 44 characters, were uniform in ornithopod evolution, or if particular branches were associated with significantly higher or lower rates of character change. In Claddis, rates are assessed by first time-scaling a phylogenetic tree, and estimating ancestral character states. Likelihood ratio tests (LRTs) are then used to test the hypothesis that a particular branch shows significantly higher or lower rates than the pooled rate for the rest of the tree, based on the total numbers of character changes and branch durations^53^,^54^. An alpha threshold of 0.01 was used to assess significance, with Benjamini-Hochberg false discovery rate correction.

Rates calculations were performed on five random MPTs out of the 190 originally calculated for the supertree. A random number generator was used for this purpose, and the selected trees were MPTs 22, 49, 50, 125 and 163. Firstly, the individual MPTs were pruned to remove non-ornithopods and taxa with excessive missing data (as described above for disparity analyses), leaving 89 tips. Stratigraphically calibrated branch lengths were calculated using the equal method^29^,^55^ by assigning taxa an age drawn randomly between their first appearance dates (FAD) and last appearance dates (LAD). For each MPT, the dating was repeated 20 times to test for consistency. To summarize rates results for all dating replicates across each MPT, we use pie charts positioned along each branch to illustrate the proportion of iterations that showed significantly high or low rates. To examine rates of character change in a temporal context, we generated time-series “spaghetti” plots using the same bins as the disparity analyses. These graphs illustrate per-lineage-million-years rates of character change in each interval, and are calculated by dividing the sum of character changes by the summed duration of the branches within each time bin. This is repeated for each dating replicate of each MPT (100 topologies in total)^56^. It is important to acknowledge that such time-series rates calculation are influenced by diversity and specimen completeness in each interval – a greater number of complete fossils allows more opportunities to record character changes^49^.

### Diversity metrics

Ornithopod diversity through time was assessed with two metrics. Firstly, we simply plotted taxonomic species diversity in each of the nine time bins used in the disparity calculations. Secondly, we calculated phylogenetic diversity estimates (PDE) incorporating both taxon occurrences and ‘ghost lineages’ inferred from the ornithopod supertree. PDE were made for the same nine time intervals. To account for phylogenetic and dating uncertainties within the supertree, unresolved nodes were randomly resolved and 100 dated topologies were generated. The median of the 100 topologies was plotted along with confidence intervals based on two-tailed 95% lower and upper quantiles.

## Additional Information

**How to cite this article**: Strickson, E. *et al.* Dynamics of dental evolution in ornithopod dinosaurs. *Sci. Rep.* **6**, 28904; doi: 10.1038/srep28904 (2016).

## Supplementary Material

Supplementary Information

Supplementary Information
