# Supplementary material for: Dynamics of dental evolution in ornithopod dinosaurs
Source: Sci Rep. 2016 Jul 14;6:28904. doi: 10.1038/srep28904 (PMC4944125; doi:10.1038/srep28904)
Supplement: Supplementary Information [file srep28904-s1.pdf]

## Supplementary appendix, tables and figures for:

# Dynamics of dental evolution in ornithopod dinosaurs

Edward Strickson, Albert Prieto-Márquez, Michael J. Benton, and Thomas L. Stubbs

1. Appendix 1 - Dental character list: pages 2-7

2. Supplementary tables: pages 8-10

3. Supplementary figures: pages 11-17

---

## Appendix 1. Dental character list

**Prieto-Márquez (2010) characters – dentary and maxillary dentition:**

**Character 1 (DTTH1).** Maximum number of tooth positions in the dentary dental battery: (0): 30 or less (mean of 22 alveolar positions); (1): 31 to 42 (mean of 37 alveolar positions); (2): more than 42 (mean of 49 alveolar positions).

**Character 2 (DTTH2).** Tooth density, expressed as the number of alveolar positions per centimeter of dental battery: (0): up to a mean density of 1.1 teeth per centimeter of dental battery (mean of 0.9); (1): more than 1.1 teeth per centimeter of dental battery (mean of 1.3).

**Character 3 (DTTH3).** Minimum number of teeth per alveoli arranged dorsoventrally at mid length of the dental battery: (0): two; (1): three; (2): four; (3): five or more. (4) one (added).

**Character 4 (DTTH4).** Maximum number of functional teeth exposed on the dentary occlusal plane (Fig. 20): (0): one; (1): one functional tooth rostrally and caudally and up to two teeth at and approaching the middle of the dental battery; (2): three functional teeth throughout most of the dental battery length, gradually changing to two near the rostral and caudal ends of the dentary.

**Character 5 (DTTH5).** Height/width ratio of the dentary tooth crowns in lingual aspect: (0): up to 1.93 (mean ratio of 1.6); (1); more than 1.93 but less than 2.7 (mean ratio of 2.4); (2): from 2.7 to 3.2 (mean ratio of 3.0); (3): more than 3.2 (mean ratio of 3.7).

**Character 6 (DTTH6).** Maximum number of ridges on the enameled lingual side of dentary tooth crowns: (0): presence of a primary major ridge extending from the ventral to the dorsal end of the crown, a rostral and slightly shorter secondary ridge and several (three or more) subdisidary, faintly developed and short tertiary ridges; (1): presence of primary, secondary, and one or two tertiary ridges; (2): presence of a primary ridge and one or two faint and shorter ridges; (3): loss of all but the primary ridge. (4) pre-development of ridge (added).

**Character 7 (DTTH7).** Dentary tooth crowns, position of the primary ridge: (0): well offset caudally from the midline; (1): median for most teeth, with some teeth within the same dentition displaying a slight caudal offset of the primary ridge.

**Character 8 (DTTH8).** Shape of the primary ridge of dentary tooth crowns: (0): straight in all teeth within the same dentition; (1): straight for some crowns and sinuous for others.

**Character 9 (DTTH9).** Angle between the crown and the root of dentary teeth: (0): up to 110º (mean angle of approximately 105º), curved root (0); (1): more than 110º and up to 135º (mean angle of approximately 125º), straight root; (2): more than 135º (mean angle of approximately 140º), straight root.

**Character 10 (DTTH10).** Overall morphology of the dentary marginal denticles: (0): wedge to tongue-shaped; (1): curved and mammillated asymmetrical ledge; 61 (2): absent or much reduced to small papillae along the apical half of the dorsal half of the crown.

**Character 11 (DTTH11).** Structure of the dentary marginal denticles: (0): each denticle contains two, three or more indentations in its apical margin; (1): each denticle is composed of three separate and rounded knobs aligned labiomesially; (2): each denticle consists of a single and rounded knob.

**Character 12 (DTTH12).** Differences in denticulation between the mesial and distal crown margins: (0): both mesial and distal margins have approximately the same quantity of denticles; the size of the denticles in both carinae is also similar. (1): the mesial margin has larger and either more or less denticles than the distal one; when that occurs, crowns tend to appear imbricated, so that the mesial margin overlaps the distal margin of the adjacent crown.

**Character 13 (DTTH13).** Thickness of the septa that separate the dentary alveolar sulci: (0): finger-like septa, as thick as the adjacent alveolar sulci or, at least, thicker than half the width of the adjacent alveolus; (1): sheet-like septa, thinner than half the width of the adjacent alveolar sulci. (2) isolated teeth (added).

**Character 14 (DTTH14).** Morphology of the alveolar sulci: (0): shaped by dentary crowns; (1): narrow and parallel-sided sulci. 2. isolated teeth (added)

**Character 15 (DTTH15).** Distribution of the enamel on dentary crowns: (0): presence of a thin veneer labially, thick lingually; (1): only present lingually.

**Character 16 (MXTH1).** Maximum number of tooth positions in the maxillary dental battery: (0): up to 33 tooth positions (mean of 23 teeth); (1): more than 33 and up to 41 tooth positions (mean of 40 teeth); (2): more than 41 tooth positions (mean of 49 teeth).

**Character 17 (MXTH2).** Number of maxillary teeth per centimeter of dental battery: (0): less than 1.27 teeth per cm of dental battery (mean ratio of 1.05); (1): 1.27 or more teeth per cm of dental battery (mean ratio of 1.44).

**Character 18 (MXTH3).** Increase in the number of tooth positions in the maxilla relative to the dentary: (0): absent; (1): present, the maxillary dental battery has from 5 to 20% more tooth positions than the dentary one.

**Character 19 (MXTH4).** Maximum number of functional teeth, per alveolar position, forming the maxillary occlusal plane: (0): one; (1): one tooth for most of the dental battery, with the sporadic presence of a second tooth forming the occlusal plane. (2): two functional teeth throughout most of the dental battery length, gradually changing to one near the rostral and caudal ends of the dentary.

**Character 20 (MXTH5).** Maximum number of ridges on the enameled labial side of maxillary tooth crowns: (0): presence of a primary major ridge and three or more much fainter ridges. (1): loss of all but the primary ridge in all or, at least, most of the crowns (in the latter situation a few crowns show a fainter secondary ridge).

**Character 21 (MXTH6).** Maxillary tooth crowns, position of the primary ridge: (0): the dental battery contains a mixture of teeth with primary ridge positioned caudally and teeth with the ridge at the center of the crown; 63 (1): the majority of teeth in the dental battery have a centered primary ridge.

**Character 22 (MXTH7).** Shape of the primary ridge of maxillary tooth crowns: (0): straight in all teeth within the same dentition; (1): straight for some crowns and sinuous for others.

**Character 23 (DTMX8).** Overall morphology of the maxillary marginal denticles: (0): wedge to tongue-shaped; (1): curved and mammillated asymmetrical ledge; (2): absent or reduced to small papillae along the apical half of the dorsal half of the crown.

**Norman (2015) characters:**

**Character 1:** Wear facet distribution on dentary and maxillary crowns: irregular and discontinuous distribution on individual crowns (0), wear facets continuous across adjacent crowns, producing a uniformly narrow cutting surface (1), oldest and other successional crowns contribute to the wear surface to varying degrees, thereby forming a transversely broad cutting/grinding occlusal surface (2).

**Character 2:** Relative crown width: maxillary crowns equal in width to dentary crowns (0), narrower than dentary crowns (1), equal in width to dentary crowns, but ‘miniaturized’ (2).

**Character 3:** Tooth roots: tapering cylinders (0), longitudinally grooved to accommodate relatively closely packed teeth (1), highly angular-sided (hexagonally prismatic) roots that indicate close packing of the teeth to form a functionally integrated polytooth magazine (2)

**Character 4:** Dentary teeth, crown shape in lingual view: coarse beech leaf-shaped profile (0), broad and shieldlike (1), coronal margin, in unworn examples, is truncated and exhibits a distinct 'shoulder' mesial to the tip of the crown formed at the intersection of the primary ridge with the coronal margin (2), mesiodistally compressed, mesial and distal coronal and apical margins converge and create an approximately diamond-shaped outline for the exposed, enamelled, tooth surface (3), curved caniform tooth shape (4) (added).

**Character 5:** Dentary teeth, crown shape 2: the midline axis of the crown in lingual view is straight (0), the entire enamelled crown face is inclined posterodorsally (1), the upper half of the crown face is distally recurved (2).

**Character 6:** Dentary teeth, presence of oblique, thickened inrolled ridges along the lower (apical) margins of the enamelled lingual face: absent (0), present (1), reduction of the rolled ridges to form a simple, thickened enamelled edge (2).

**Character 7:** Dentary teeth size, relative to the alveolar trough: small and leaf-shaped in profile (0), large and shield-shaped (1), miniaturized (2), large and curved (3) (added).

**Character 8:** Maxillary teeth shape: approximately equal in width to dentary crowns (0), narrower and more lanceolate than opposing crowns (1), lanceolate and equal in width to opposing dentary crowns (2).

**Butler (2011) characters:**

**Character 1:** Premaxillary teeth, number: (0). Six; (1) Five; (2) Four; (3) Three; (4) Two; (5) One. (6) None (added).

**Character 2:** Premaxillary teeth, crown expanded above root: (0) Crown is unexpanded mesiodistally above root, no distinction between root and crown is observable; (1) Crown is at least moderately expanded above root. (2) None (added).

**Character 3:** Premaxillary teeth increase in size posteriorly: (0) Absent, all premaxillary teeth subequal in size; (1) Present, posterior premaxillary teeth are significantly larger in size than anterior teeth. (2) None (added).

**Character 4:** Maxillary/dentary teeth, marginal ornamentations: (0) Fine serrations set at right angles to the margin of the tooth; (1) Coarse serrations (denticles) angle upwards at 45 degrees from the margin of the tooth. (2) No visible serrations (added)

**Character 5:** At least moderately developed labiolingual expansion of crown ('cingulum') on maxillary/dentary teeth: (0) Present; (1) Absent.

**Character 6:** Heterodont dentary dentition: (0) No substantial heterodonty is present in dentary dentition; (1) Single, enlarged, caniform anterior dentary tooth, crown is not mesiodistally expanded above root; (2) Anterior dentary teeth are strongly recurved and caniform, but have crowns expanded mesiodistally above their roots and are not enlarged relative to other dentary teeth.

**Character 7**: Peg-like tooth located anteriorly within dentary, lacks denticles, strongly reduced in size: (0) Absent; (1) Present.

**Character 8:** Recurvature in maxillary and dentary teeth: (0) Present; (1) Absent.

**Character 9:** Overlap of adjacent crowns in maxillary and dentary teeth: (0) Absent; (1) Present.

**Character 10:** Crown is mesiodistally expanded above root in cheek teeth: (0) Absent; (1) Present.

**Character 11:** Position of maximum apicobasal crown height in dentary/maxillary tooth rows: (0) Anterior portion of tooth row; (1) Central portion of tooth rows; (2) Caudal portion of tooth rows.

**Character 12:** Dentary: Close-packing and quicker replacement eliminates spaces between alveolar border and crowns of adjacent functional teeth: (0) Absent; (1) Present.

**Character 13.** Maxilla: Close-packing and quicker replacement eliminates spaces between alveolar border and crowns of adjacent functional teeth: (0) Absent; (1) Present.

**10 PC axes NPMANOVA**
*F*: 9.994 – $p < 0.0001$

| | Jeholosaurid | Pre-Igua and Post-Jehol | Rhabdodontid | Post-Rhab and Dryosaurids | Ankylo-Basal Styraco | Styracosterns | Hadrosauroid | Hadrosaurid | Lambeo | Saurolo |
|---|---|---|---|---|---|---|---|---|---|---|
| Jeholosaurid | | 0.4147 | **0.0180** | **0.0340** | **0.0114** | **0.0023** | **0.0010** | **0.0002** | **0.0002** | **0.0006** |
| Pre-Igua and Post-Jehol | 0.4147 | | **0.0114** | **0.0180** | **0.0046** | **0.0023** | **0.0002** | **0.0002** | **0.0002** | **0.0002** |
| Rhabdodontid | **0.0152** | **0.0091** | | 0.2917 | 0.0587 | **0.0010** | **0.0009** | **0.0002** | **0.0004** | **0.0002** |
| Post-Rhab and Dryosaurids | **0.0295** | **0.0151** | 0.2852 | | 0.0888 | **0.0027** | **0.0008** | **0.0004** | **0.0002** | **0.0002** |
| Ankylo-Basal Styraco | **0.009** | **0.0033** | 0.0522 | 0.0829 | | **0.0114** | **0.0049** | **0.0002** | **0.0002** | **0.0002** |
| Styracosterns | **0.0014** | **0.0015** | **0.0006** | **0.0018** | **0.0088** | | **0.0028** | **0.0002** | **0.0002** | **0.0002** |
| Hadrosauroid | **0.0006** | **0.0001** | **0.0005** | **0.0004** | **0.0036** | **0.0019** | | **0.0002** | **0.0002** | **0.0002** |
| Hadrosaurid | **0.0001** | **0.0001** | **0.0001** | **0.0002** | **0.0001** | **0.0001** | **0.0001** | | 0.1086 | 0.0655 |
| Lambeo | **0.0001** | **0.0001** | **0.0002** | **0.0001** | **0.0001** | **0.0001** | **0.0001** | 0.1038 | | **0.0002** |
| Saurolo | **0.0003** | **0.0001** | **0.0001** | **0.0001** | **0.0001** | **0.0001** | **0.0001** | 0.0597 | **0.0001** | |

**28 PC axes NPMANOVA**
*F*: 4.606 – $p < 0.0001$

| | Jeholosaurid | Pre-Igua and Post-Jehol | Rhabdodontid | Post-Rhab and Dryosaurids | Ankylo-Basal Styraco | Styracosterns | Hadrosauroid | Hadrosaurid | Lambeo | Saurolo |
|---|---|---|---|---|---|---|---|---|---|---|
| Jeholosaurid | | 0.3585 | **0.0187** | 0.0622 | **0.0139** | **0.0029** | **0.0007** | **0.0002** | **0.0002** | **0.0002** |
| Pre-Igua and Post-Jehol | 0.3426 | | **0.0187** | **0.0185** | **0.0030** | **0.0007** | **0.0004** | **0.0002** | **0.0002** | **0.0002** |
| Rhabdodontid | **0.0158** | **0.0157** | | 0.2952 | 0.0570 | **0.0018** | **0.0004** | **0.0002** | **0.0002** | **0.0004** |
| Post-Rhab and Dryosaurids | 0.0553 | **0.0148** | 0.2755 | | 0.2355 | **0.0065** | **0.0030** | **0.0002** | **0.0002** | **0.0002** |
| Ankylo-Basal Styraco | **0.0108** | **0.0021** | **0.0494** | 0.2146 | | **0.0044** | **0.0017** | **0.0002** | **0.0002** | **0.0002** |
| Styracosterns | **0.0019** | **0.0004** | **0.0011** | **0.0049** | **0.0031** | | **0.0053** | **0.0002** | **0.0002** | **0.0002** |
| Hadrosauroid | **0.0004** | **0.0002** | **0.0002** | **0.0021** | **0.001** | **0.0039** | | **0.0002** | **0.0002** | **0.0002** |
| Hadrosaurid | **0.0001** | **0.0001** | **0.0001** | **0.0001** | **0.0001** | **0.0001** | **0.0001** | | 0.5506 | 0.3607 |
| Lambeo | **0.0001** | **0.0001** | **0.0001** | **0.0001** | **0.0001** | **0.0001** | **0.0001** | 0.5506 | | **0.0002** |
| Saurolo | **0.0001** | **0.0001** | **0.0002** | **0.0001** | **0.0001** | **0.0001** | **0.0001** | 0.3527 | **0.0001** | |

**Supplementary table 1.** NPMANOVA results testing for group separation in ornithopod dental morphospace. The distribution of the ten major groupings is compared, based on the first 10 PC axes and the first 28 PC axes (95% variance). Uncorrected (below diagonal) and

false discovery rate corrected (above diagonal) *p*-values are reported. *p*-values $< 0.05$ denote significantly different morphospace occupation. The taxon-sorted bins in full are: ‘jeholosaurids’, ‘pre-iguanodontid/post-jeholosaurid’, ‘rhabdodontids’, ‘post-rhabdodontids and dryosaurids’, ‘basal ankylopollexians and basal styracosterns’, ‘basal styracosterns’, ‘basal hadrosauroids’, ‘hadrosaurids’, ‘lambeosaurines’, and ‘saurolophines’.

**10 PC axes NPMANOVA**
$F$: 3.507 – $p < 0.0001$

| | Kim-Tth | Ber-Vlg | Hau-Bar | Aptian | Albian | Cen-Tur | Con-San | Cmp | Maa |
|---|---|---|---|---|---|---|---|---|---|
| Kim-Tth | | 0.5859 | 0.7503 | 0.6056 | 0.5859 | 0.7503 | 0.5859 | **0.0082** | 0.0714 |
| Ber-Vlg | 0.4069 | | 0.5859 | 0.5369 | 0.6056 | 0.8301 | 0.4310 | **0.0133** | 0.1793 |
| Hau-Bar | 0.686 | 0.3788 | | 0.8999 | 0.5859 | 0.7014 | 0.4553 | **0.0009** | **0.0104** |
| Aptian | 0.4542 | 0.2983 | 0.8999 | | 0.8999 | 0.6399 | 0.0869 | **0.0009** | **0.0036** |
| Albian | 0.3551 | 0.4542 | 0.3624 | 0.8807 | | 0.7014 | 0.0714 | **0.0009** | **0.0036** |
| Cen-Tur | 0.6878 | 0.784 | 0.604 | 0.4977 | 0.5945 | | 0.7014 | **0.0009** | 0.0869 |
| Con-San | 0.3981 | 0.2155 | 0.2403 | **0.0338** | **0.0238** | 0.591 | | **0.0120** | 0.1793 |
| Cmp | **0.0016** | **0.0037** | **0.0001** | **0.0001** | **0.0001** | **0.0001** | **0.003** | | 0.3621 |
| Maa | **0.0233** | 0.0781 | **0.0023** | **0.0005** | **0.0006** | **0.0332** | 0.0797 | 0.171 | |

**28 PC axes NPMANOVA**
$F$: 2.286– $p < 0.0001$

| | Kim-Tth | Ber-Vlg | Hau-Bar | Aptian | Albian | Cen-Tur | Con-San | Cmp | Maa |
|---|---|---|---|---|---|---|---|---|---|
| Kim-Tith | | 0.3620 | 0.5676 | 0.5072 | 0.2404 | 0.5082 | 0.2045 | **0.0062** | **0.0408** |
| Ber-Vlg | 0.2011 | | 0.5435 | 0.3587 | 0.5537 | 0.5435 | 0.3686 | **0.0112** | 0.1357 |
| Hau-Bar | 0.4888 | 0.4363 | | 0.9642 | 0.6316 | 0.5435 | 0.4088 | **0.0012** | **0.0081** |
| Aptian | 0.3381 | 0.1893 | 0.9642 | | 0.9642 | 0.5435 | 0.1390 | **0.0012** | **0.0018** |
| Albian | 0.1135 | 0.4614 | 0.5614 | 0.9584 | | 0.7977 | 0.1358 | **0.0012** | **0.0036** |
| Cen-Tur | 0.3529 | 0.4072 | 0.4376 | 0.4378 | 0.7534 | | 0.7548 | **0.0022** | **0.0311** |
| Con-San | 0.0909 | 0.215 | 0.2498 | 0.0579 | 0.0528 | 0.6919 | | **0.0216** | 0.2776 |
| Cmp | **0.0012** | **0.0028** | **0.0001** | **0.0001** | **0.0001** | **0.0003** | **0.006** | | 0.4876 |
| Maa | **0.0136** | **0.049** | **0.0018** | **0.0002** | **0.0006** | **0.0095** | 0.1388 | 0.3115 | |

**Supplementary table 2.** Tests for significant shifts/differences in the distribution of ornithopods in dental morphospace between time bins. Pairwise tests are based on non-parametric multivariate analysis of variance (NPMANOVA), performed on PC scores from the first 10 PC axes and the first 28 PC axes (95% variance). Uncorrected (below diagonal) and false discovery rate corrected (above diagonal) *p*-values are reported. *p*-values < 0.05 denote significantly different morphospace occupation.

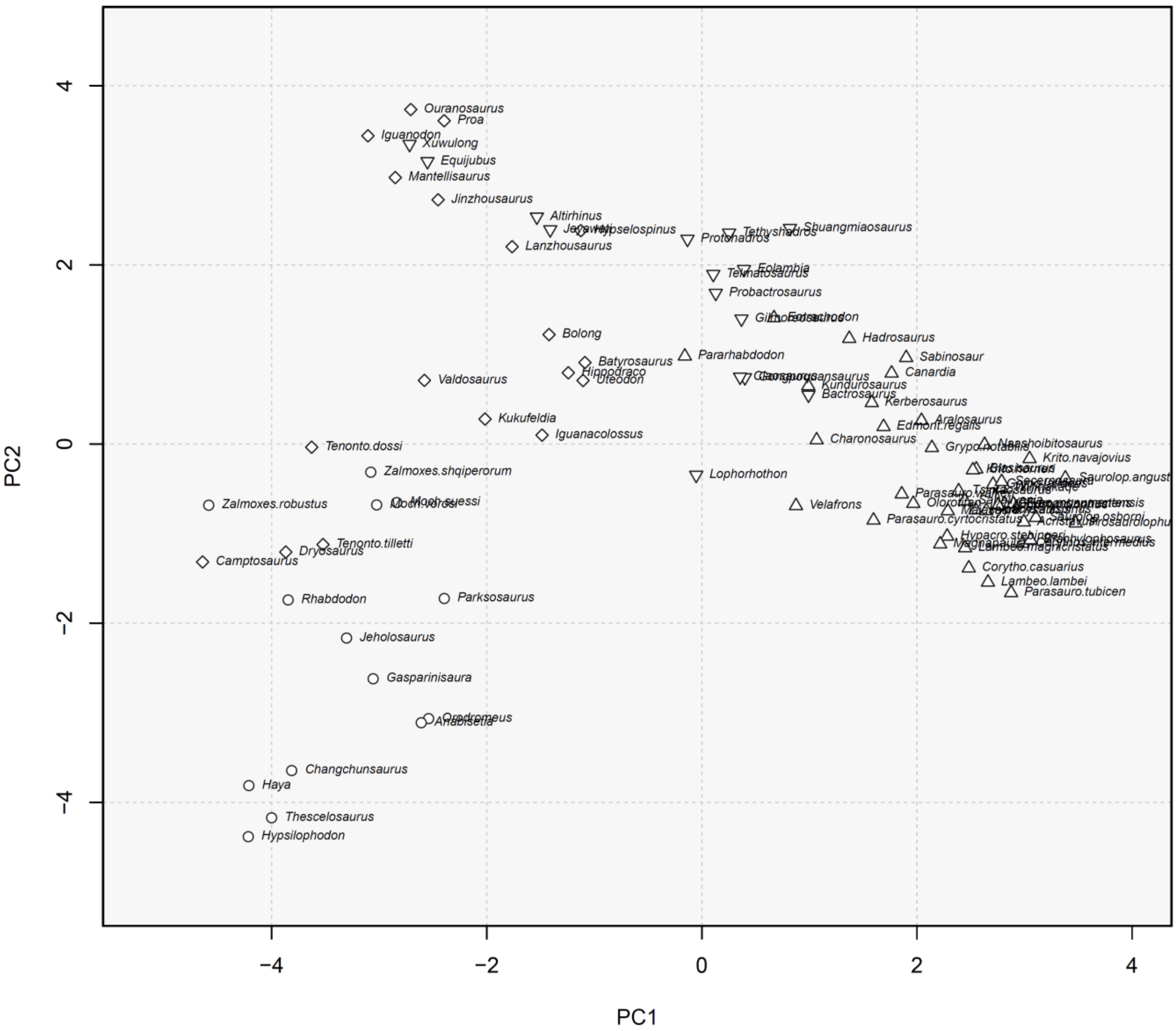


**Supplementary Figure 1.** Ornithopod dental morphospace, defined by PC1 (8.5%) and PC2 (4.2%). All taxa are individually labelled.

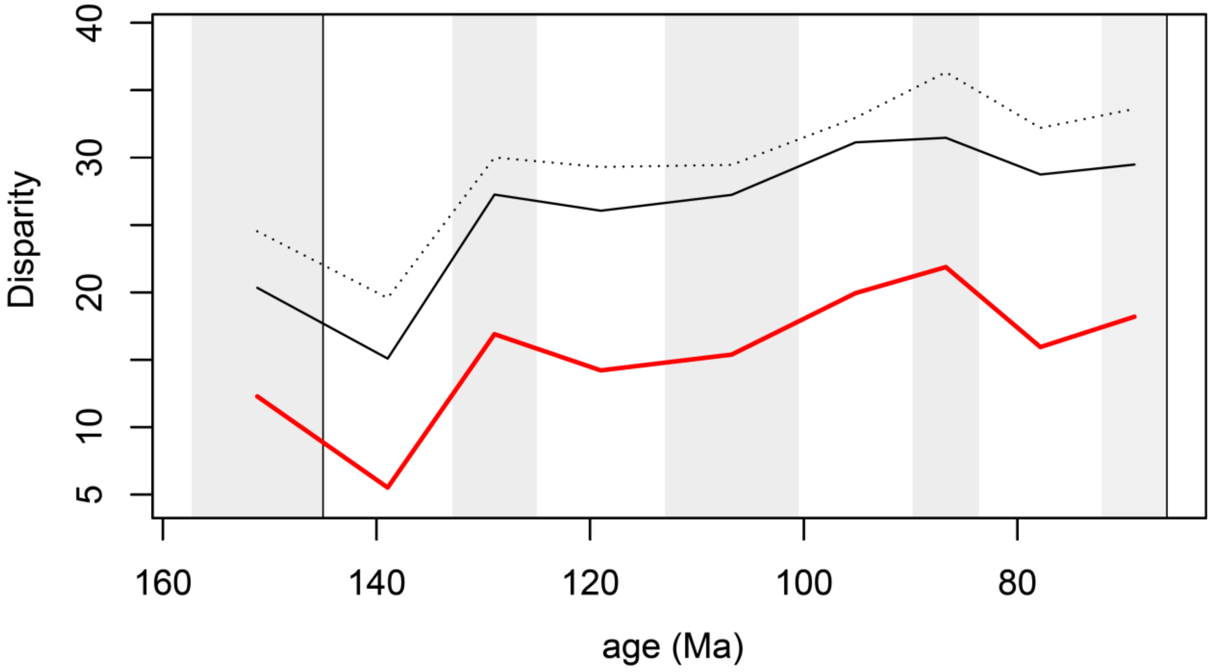


**Supplementary Figure 2.** Temporal trends of ornithopod dental disparity. Disparity based on the sum of variances metric is plotted in nine time intervals. Each curve corresponds to disparity calculated using scores from a different number of PC axes: 10 axes, as in main text figure 4.e (red), 23 axes (solid black line), and 28 axes (dashed black line). Overall, the same trend of disparity through time is recovered in all calculations, with peaks and troughs largely corresponding.

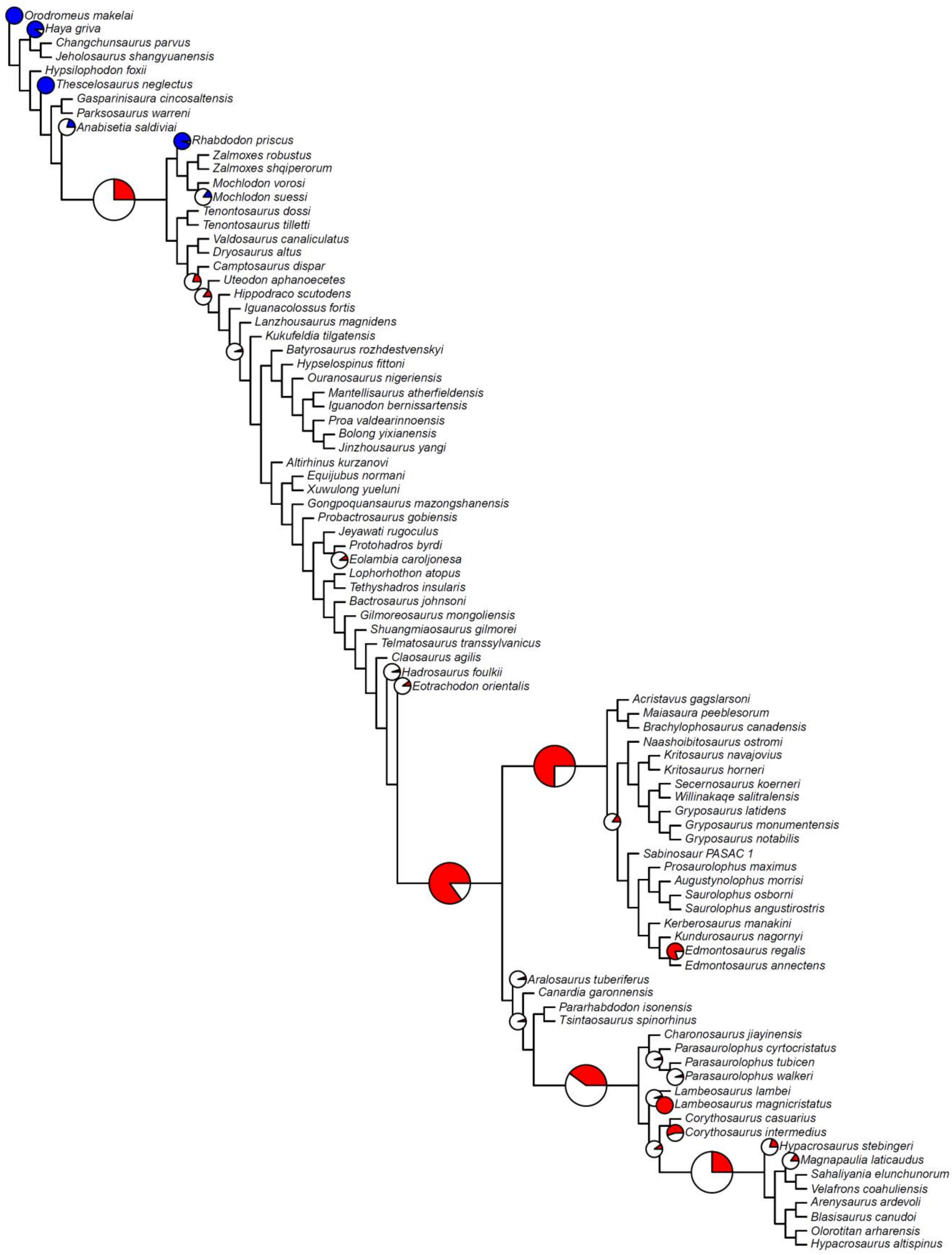


**Supplementary Figure 3. MPT 50**

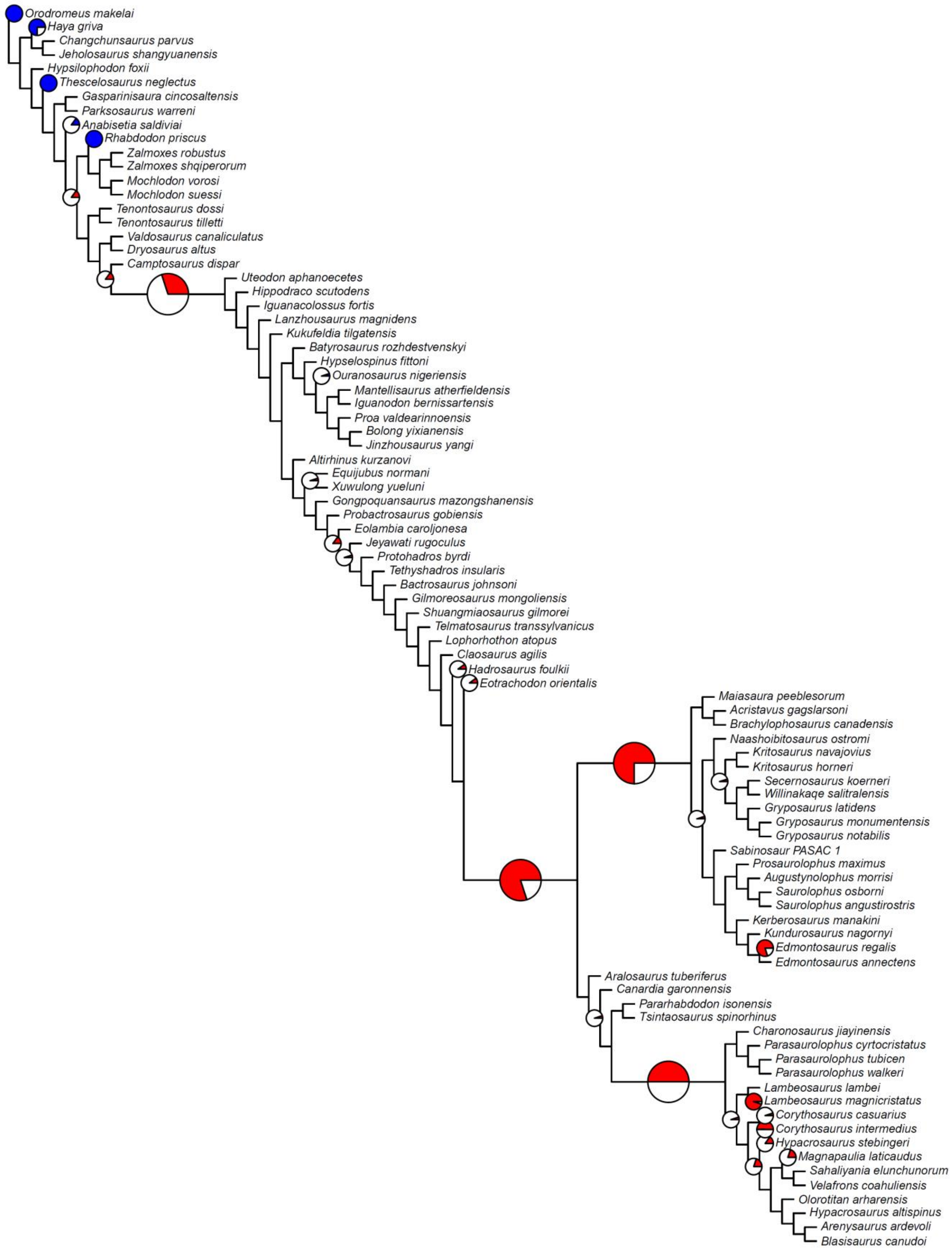


**Supplementary Figure 4. MPT 125**

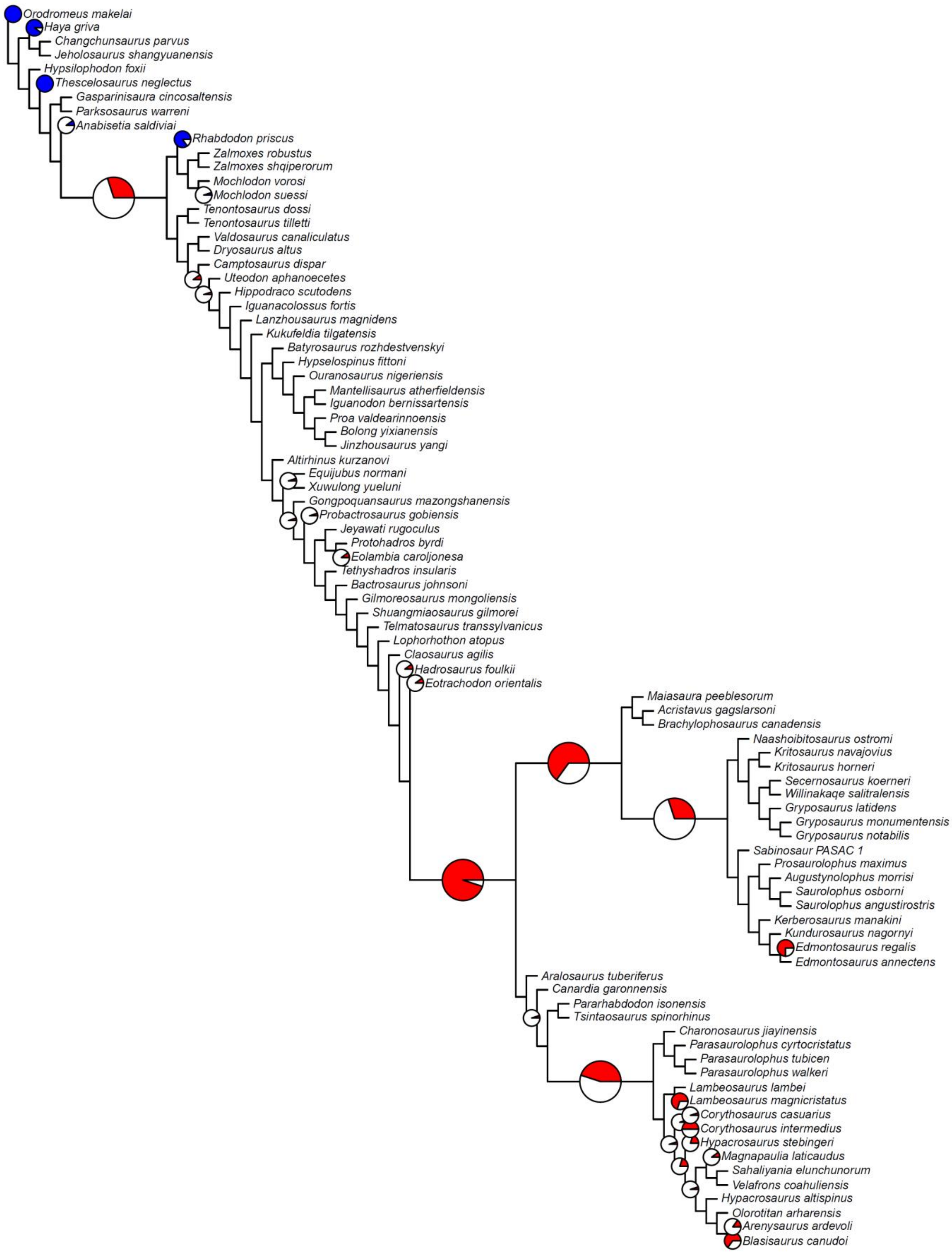


**Supplementary Figure 5. MPT 22**

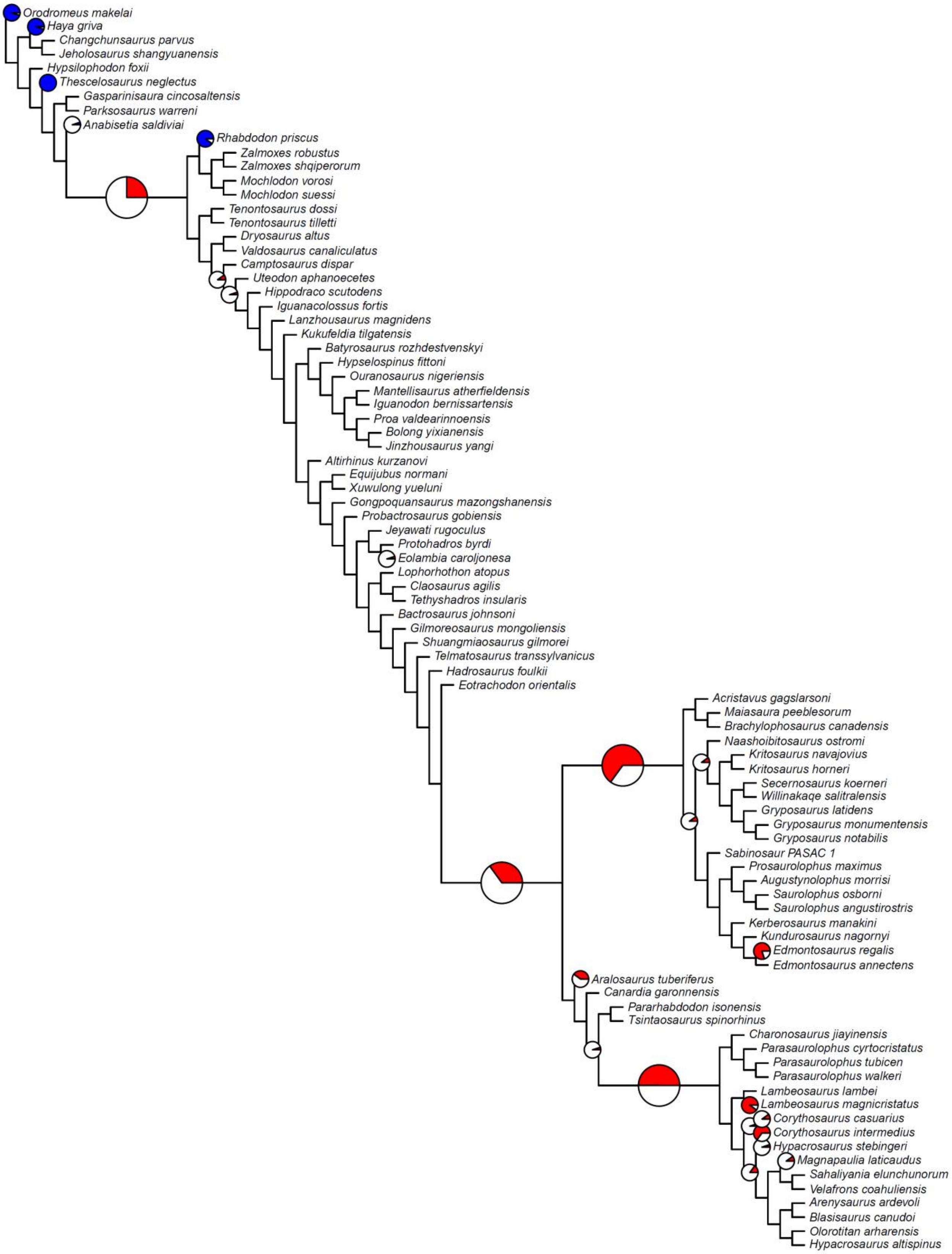


**Supplementary Figure 6. MPT 49**

**Supplementary Figures 3-6.** Rates of morphological evolution based on discrete dental characters in Ornithopoda. The proportion of significantly high (red) and significantly low (blue) per-branch rates based on 20 dating replicates are illustrated with pie charts. There are no pie charts positioned on branches which showed nonsignificant rates in all dating replicates. Internal branches which showed significantly high/low rates in more than 25% of trees are lengthened and have enlarged pie charts. Four additional MPTs are figured here, the results are generally consistent across all trees. In MPTs 50 and 22, additional branches with high rates in a large proportion of dating replicates are identified in the hadrosaurids.
